# Supplementary material for: Multimode polariton effects on molecular energy transport and spectral fluctuations
Source: Commun Chem. 2022 Apr 6;5:48. doi: 10.1038/s42004-022-00660-0 (PMC9814737; doi:10.1038/s42004-022-00660-0)
Supplement: Supplementary file 1 — Supplemental Materials [file 42004_2022_660_MOESM1_ESM.pdf]

Supplemental Material for Multimode polariton effects on intermolecular energy transport and spectral fluctuations

Raphael F. Ribeiro*
Department of Chemistry and Cherry Emerson Center for Scientific Computation, Emory University, Atlanta, GA, USA
(Dated: March 29, 2022)

CONTENTS

I. **Supplementary Note 1.** Photon density of states effect in thermodynamic limit computations with different lattice constants 1

II. **Supplementary Note 2.** Structural and energetic disorder 2

References 3

I. SUPPLEMENTARY NOTE 1. PHOTON DENSITY OF STATES EFFECT IN THERMODYNAMIC LIMIT COMPUTATIONS WITH DIFFERENT LATTICE CONSTANTS

In this section we present the behavior of the molecular excited-state return probability as a function of the mean intermolecular distance (lattice constant) a for systems with equal size $L = L_0$, and compare these results with those in the main manuscript where L varies with the lattice constant a as the number of photon modes remains constant. Our purpose is to show that the change in photon density of states

$$\rho_C(\omega) = \sum_q \delta(\omega - \omega_q), \tag{1}$$

that results when N_C is fixed while a and $L = N_C a$ are changed does not influence the effects of the cavity-matter interaction on the considered molecular observables (ω_q and the allowed discrete values of q are defined in the main text). Note that in the continuum limit,

$$\rho_C(\omega) \to \frac{L}{2\pi} \int_{-\pi/a}^{\pi/a} \mathrm{d}q \; \delta(\omega - \omega_q) \tag{2}$$

Hence, a change in a leads to a modification in the photon density of states in the thermodynamic limit that could presumably have an effect on the molecular observables we computed below. As we show below, this change has insignificant effect.

In order to show that the change in the photonic DOS associated to a variation of a with fixed $N_M = N_C$ plays an insignificant role in our comparative analysis of systems with different mean intermolecular distances, we compute, with fixed collective light-matter interaction $\Omega_R = 0.3$ eV, the ensemble-averaged molecular return probability $\Pi_M(L_0)$ for $a = 25$ and 50 nm with $N_M = N_C$ chosen such that $L = L_0 = 0.01001$ mm with varying energetic disorder σ and compare the results to the Π_M reported in the main text where $N_C = 1001$ and $L = 0.025$ mm, and 0.05 mm for $a = 25$ and 50 nm, respectively. The choice of $L_0 = 0.01$ mm as reference is motivated by the fact that this is the L obtained when $N_C = 1001$ and $a = 10$ nm (the smallest lattice constant considered in the main manuscript). Therefore, in the computations with fixed $L = L_0$, the number of degrees of freedom become smaller by factors of 2.5 and 5 in order to ensure $N_C a$ is fixed at 0.01 mm for systems with $a = 25$ and $a = 50$ nm respectively. The results obtained for systems with $N_M = N_C < 1001$ are generated from ensemble averaging over 100 realizations in each case to reduce standard deviation in the predictions for the smaller systems. See main manuscript for information about other parameters employed in the computations below (see Thermodynamic limit convergence).

* raphael.ribeiro@emory.edu

σ/Ω_R	$\Pi_M(L_0)$	Π_M
1.20	0.459 ± 0.022	0.447 ± 0.008
1.00	0.431 ± 0.022	0.426 ± 0.011
0.80	0.401 ± 0.021	0.397 ± 0.008
0.60	0.362 ± 0.016	0.358 ± 0.007
0.40	0.317 ± 0.014	0.316 ± 0.006
0.30	0.298 ± 0.01	0.298 ± 0.004
0.20	0.287 ± 0.008	0.286 ± 0.003
0.10	0.275 ± 0.009	0.274 ± 0.003
0.05	0.249 ± 0.007	0.243 ± 0.002
0.00	0.00679	0.00137

Supplementary Table I: Molecular excited-state return probabilities $\Pi_M(L_0)$ and Π_M in polaritonic systems with $a = 50$nm, $\Omega_R = 0.3$ eV, $E_C(0) = E_M = 2.0$ eV obtained with $N_M = N_C = 201$ and $N_M = N_C = 1001$ and corresponding long axis length $L_0 \approx 0.01$ mm (the same value obtained for a system with $N_M = N_C = 1001$ and $a = 10$ nm) and $L = 0.05$ mm, respectively.

σ/Ω_R	$\Pi_M(L_0)$	Π_M
1.20	0.471 ± 0.015	0.461 ± 0.01
1.00	0.442 ± 0.017	0.436 ± 0.009
0.80	0.408 ± 0.014	0.405 ± 0.007
0.60	0.367 ± 0.011	0.368 ± 0.006
0.40	0.325 ± 0.01	0.325 ± 0.005
0.30	0.311 ± 0.007	0.310 ± 0.007
0.20	0.312 ± 0.006	0.311 ± 0.003
0.10	0.317 ± 0.007	0.315 ± 0.006
0.05	0.304 ± 0.006	0.303 ± 0.005
0.00	0.00358	0.00143

Supplementary Table II: Molecular excited-state return probabilities $\Pi_M(L_0)$ and Π_M as a function of energetic disorder in polaritonic systems with $a = 25$nm, $\Omega_R = 0.3$ eV, $E_C(0) = E_M = 2.0$ eV obtained with $N_M = N_C = 401$ and $N_M = N_C = 1001$ and corresponding long axis length $L_0 \approx 0.01$ mm (the same value obtained for a system with $N_M = N_C = 1001$ and $a = 10$ nm) and $L = 0.025$ mm, respectively.

Tables 1 and 2 show that while models with distinct N_C and fixed a give rise to differences in the thermodynamic limit photon density of states (Eq. 2), there is no appreciable effect of this variation on the molecular excited-state return probability, except when there is no disorder in which case all eigenstates extend over L and the molecular return probability with fixed a has a simple $1/L$ scaling.

II. SUPPLEMENTARY NOTE 2. STRUCTURAL AND ENERGETIC DISORDER

As described in the main text, we have also introduced heterogeneity in the molecular transition dipole moments by sampling these quantities from a normal distribution with variance σ_μ. When energetic disorder σ is small relative to Ω_R, structural disorder provides a mechanism for the localization of polaritonic and weakly coupled ("dark") excitations.

In Supplementary Figure 1, we verify this result by showing the cavity-induced change in the molecular local DOS entropy $\Delta S[\rho_M]$ and excitation escape probability χ_M for systems with $N_M = N_C = 1001$, $a = 10$ nm, $E_M = E_C(0) = 2.0$ eV, $\Omega_R = 0.3$ eV and varying levels of energetic and structural disorder. It follows from these results that, as expected, structural disorder becomes essential when the energetic disorder is insignificant. However, we also infer from Supplementary Figure 1 that the optical cavity effect on both $\Delta S[\rho_M]$ and χ_M is much more sensitive to the energetic disorder strength than it is to static dipole magnitude fluctuations.

Curiously, while energetic and dipolar disorder act to localize molecular excited-states as measured by χ_M, they have opposing effects on $\Delta S[\rho_M]$ decreasing with energetic disorder, but increasing with σ_μ. This behavior can be understood by noting that energetic disorder primarily drives strong localization of states with a large molecular content (Fig. 5 of main text) via its introduction of traps for excitation migration. This also leads to a reduction in $\Delta S[\rho_M]$, since for fixed σ_μ "dark states" of systems with greater σ share greater similarity with bare modes as σ increases. Energetic disorder also localizes polariton modes by mixing of excitations with distinct wave-vectors q. Conversely, while an increase in the magnitude of fluctuations of the molecular transition dipole moment also leads to polariton localization by mixing modes with distinct values of q, an increase of σ_μ leads to stronger fluctuations in the magnitude of light-matter interactions, as the coupling constant of the jth molecule interaction with the cavity mode q is proportional to $\mu_j \sqrt{\omega_M/\omega_q}$. This manifests into an effective increase in the strength of the *collective* (resonant) light-matter interaction squared which becomes approximately equal to $\Omega_R^2 \left(1 + \sigma_\mu^2/\mu_0^2\right)$. Hence, the observed Rabi splitting is stronger in systems with larger dipolar fluctuations with their resulting LP and UP excitations separated by greater magnitude from the bare ensemble excitation energies. It follows that inside an optical cavity, a molecular ensemble with greater fluctuation in transition dipole magnitude will be able to access a wider range of energies and

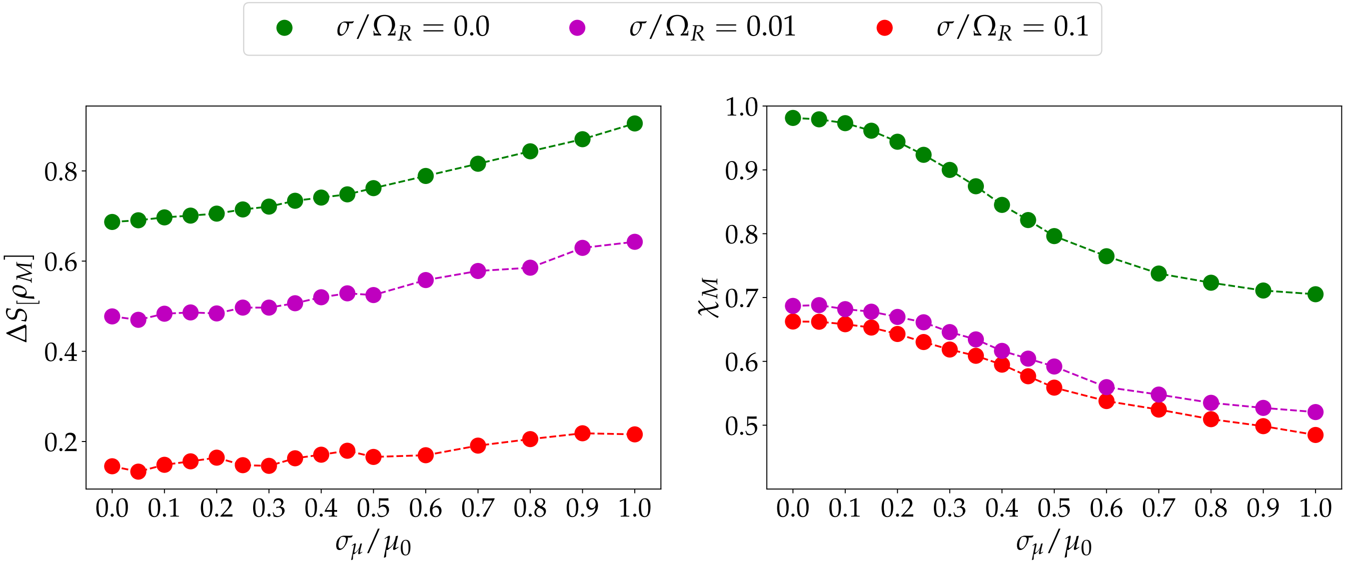

Supplementary Figure 1: Dipolar disorder effect on $\Delta S[\rho_M] = S[\rho_M] - S[\rho_M^{(0)}]$ (left) and $\chi_M = 1 - \Pi_M$ (right) for varying levels of energetic and structural disorder in systems with $N_M = N_C = 1001$, $E_0 = E_C(0) = 2.0$ eV, $\Omega_R = 0.3$ eV. Each data point corresponds to the average result obtained from 10 realizations.

therefore $\Delta S[\rho_M]$ will increase with σ_μ as evidenced in Supplementary Figure 1.
